# Adolescent extracurricular activities and perception of risk of harm from binge drinking

**DOI:** 10.1371/journal.pmen.0000278

**Published:** 2025-04-09

**Authors:** Claire Szapary, Jenny Meyer, Claudia-Santi F. Fernandes, Tyra Pendergrass Boomer, Lynn Fiellin, Kammarauche Aneni

**Affiliations:** 1 Yale School of Public Health, New Haven, Connecticut, United States of America; 2 Department of Internal Medicine, Yale School of Medicine, New Haven, Connecticut, United States of America; 3 Yale Child Study Center, Yale School of Medicine, New Haven, Connecticut, United States of America; 4 Biomedical Informatics and Data Science, Yale School of Medicine, New Haven, Connecticut, United States of America; 5 Dartmouth Geisel School of Medicine, Hanover, New Hampshire, United States of America; 6 Biomedical Data Science, Dartmouth Geisel School of Medicine, Hanover, New Hampshire, United States of America; UCL: University College London, UNITED KINGDOM OF GREAT BRITAIN AND NORTHERN IRELAND

## Abstract

Underage binge drinking can lead to numerous adverse health consequences. Preventing excessive alcohol consumption by targeting perception of risk of harm is one approach with demonstrated success. This study examined the association between participation in extracurricular activities and perception of risk of harm from binge drinking among adolescents. A cross-sectional analysis of adolescents aged 12-17 years was performed using the 2019 National Survey on Drug Use and Health dataset. Participation in any extracurricular activity as well as four types of extracurricular activities (e.g., school-based, community-based, faith-based, and other) were the main independent variables. The primary outcome was perception of risk of harm from weekly binge drinking. Bivariate and multivariate logistic regression models were used to examine this association. Of the 11,870 adolescents included in the analysis, 4.2% reported that weekly binge drinking presented no harm. Compared to adolescents who did not participate in any extracurricular activity, adolescents who participated in one or more extracurricular activity, regardless of type, had 86% higher odds of reporting risk of harm from weekly binge drinking after adjusting for relevant covariates (aOR=1.86, 95% CI: 1.39, 2.49). This finding was similar across all levels of school-based, but different for community, faith-based and other types of activities. Compared to adolescents who did not participate in school-based, adolescent who participated in 1, 2, or 3+ school-based activities were more likely to report that weekly binge drinking was harmful (*School* AOR 1 activity = 1.75, 95% CI: 1.29, 2.37, AOR 2 activities = 1.83, 95% CI:1.32, 2.53, AOR 3+ activities = 1.91, 95% CI:1.35, 2.75), The association between participation in community-based activities and perception of risk of harm was significant at one or two activities but not at three or more activities (*Community* AOR 1 activity = 1.35, 95% CI: 1.02, 1.79, AOR 2 activities = 1.47, 95% CI:1.06, 2.02, AOR 3+ activities = 1.41, 95% CI: 0.98, 2.02) while the association between participation in faith-based activities and perception of risk of harm from weekly binge drinking was significant only at 3+ activities (*Faith* AOR 3+ activities = 2.07, 95% CI: 1.48, 2.88). No significant association was found for participation in other activities in the adjusted model. Our results suggest that participation in extracurricular activities may be a factor that contributes to increased perception of risk of harm from weekly binge drinking among adolescents, but this protective effect may vary by type and level of activity, suggesting different mechanistic pathways. Future studies are needed to further elucidate these findings to inform targeted preventive interventions and policy-level support.

## Introduction

Alcohol is one of the most commonly misused substances among adolescents, presenting a serious public health problem [1]. When adolescents drink, they often consume alcohol in large and dangerous amounts, otherwise known as binge drinking (i.e., having five or more drinks of an alcoholic beverage once or twice a week) [1,2]. In fact, more than 90% of alcoholic beverages consumed by adolescents are through binge drinking [1]. Not only does this pose the risk of legal repercussions, but binge drinking can also cause physical injury and death [3], interfere with the development of brain structure and function [1], and increase the likelihood of developing alcohol use disorder later in life [4]. Despite these concerns, many adolescents still engage in binge drinking. These issues highlight the need to understand factors that contribute to problematic drinking among adolescents to develop and implement preventive solutions.

One factor that may predispose adolescents to binge drinking is the lack of perception that this behavior poses a risk to them [5]. Typically, those who perceive binge drinking to be low-risk consume alcohol at a higher rate than their peers who perceive binge drinking to be risky [6–8]. Prevention intervention programs target perception of risk of harm of substances, including alcohol, as a mechanism through which to reduce the likelihood of use [9,10]. Prevention programs have been shown to effectively increase adolescents knowledge about the risks associated with substance use, which subsequently increases their risk perception [11]. A better understanding the factors that are associated with increased perception of risk of problematic alcohol use among adolescents can inform and expand prevention targets.

Engaging in extracurricular activities has been associated with improved health and functional outcomes among adolescents [12,13]. Schools generally offer extracurricular activities, making them a potentially accessible protective factor for adolescents. Outside of school, adolescents may also be involved in extracurricular activities within their faith or community settings. The protective effect of extracurricular activities on marijuana and illicit drugs use has been documented [14,15]. However, studies examining the association between participation in extracurricular activities and problematic alcohol use have revealed mixed results, suggestive of a complex association [16]. While some studies have shown a positive association between participation in sports and alcohol use [14,17–18], other research has demonstrated either a protective effect against problematic alcohol use [19] or no association at all [20–22]. In an attempt to disentangle these findings, existing studies have noted that the type of sport (individual versus team), the culture of the particular team, existence of other comorbid problem behaviors, and age of adolescent may partially explain the observed differences [16,17,19]. The majority of the studies examining the association between extracurricular activities and problematic alcohol use have focused on school-based activities. Among the limited research measuring participation in faith-based and community-based extra-curriculars, such as youth groups and community service, findings revealed lower odds of experimentation with drinking [23–25]. These differences suggest that the type of extracurricular activity may also play a role in protecting against problematic alcohol use and underscore the need for further research to elucidate this relationship. While some of these studies have shown a protective effect of participation in extracurricular activities on binge drinking, the mechanistic pathways remain unclear.

Given the complex relationship between extracurricular activity participation and problematic alcohol use and the role that risk perception can play in reducing the likelihood of future problematic drinking, this current study sought to investigate whether there is an association between participation in extracurricular activities and perception of risk of harm from binge drinking. Related studies on alcohol use suggest that extracurricular activity participation often leaves less unscheduled time for adolescents to drink alcohol [26] and facilitates the forging of relationships with mentors and adults who might serve as role models for healthy behaviors regarding substance use [26]. In addition, many schools have policies that outline consequences of drug use that can impact an adolescents’ participation in school-based extracurricular activities, which may increase their perception of harm from using substances [26]. However, to the best of our knowledge, the association between participation in extracurricular activity and perception of harm from binge drinking has yet to be formally examined. The objective of this study was to explore the association between extracurricular activity and perception of risk of harm from binge drinking utilizing data collected from 12-17-year-olds in the 2019 National Survey on Drug Use and Health (NSDUH) dataset. Specifically, we examined participation in any extracurricular activity, level of extracurricular activity involvement, and specific types/settings of activity (school, community, faith and other). We hypothesized that there would be a positive association between participation in extracurricular activity and risk perception of binge drinking, and that these effects would be amplified among those who participated in an increasing number of activities, after controlling for covariates.

### Theoretical frameworks

The health belief model and socio-ecological model guided this study. The health belief model posits that an individual will make choices about their health behavior based on how severe they perceive their risk of illness/injury [27]. As a characteristic of adolescence, youth may have a heightened sense of invincibility, coupled with a potential lack of knowledge about the risks associated with binge drinking—potentially increasing their risk of the behavior [28]. Several studies have documented that adolescents who perceive risk or susceptibility to alcohol use or binge drinking are less likely to drink [6–8], and alcohol preventive interventions have targeted perceived susceptibility to alcohol use as one potential mechanism for influencing alcohol use [29].

The socio-ecological model proposes that the environment where an adolescent resides contributes to problematic behaviors such as alcohol use. The environment includes the following domains: individual (e.g., presence of a mental disorder, perception of risk of harm from binge drinking), peer (e.g., peer beliefs about substance use), family (e.g., parental support), and community-level (e.g., access to substances) [30,31]. The interaction of these domains rather than a single factor alone contributes to the risk for engaging in problematic behavior [32]. As such, understanding how specific factors (e.g., extracurricular activity) within their ecological domains predispose adolescents to problematic alcohol use should consider the influence of other potentially salient factors. The variables included in this study reflect the theoretical underpinnings of each of these conceptual frameworks.

## Methods

### Ethics

This study was deemed exempt human subjects research by the Yale Institutional Review Board (IRB) and Research Ethics Board (REB) #2000036003. The data used in this study is publicly available data from the National Survey on Drug Use and Health (NSDUH) (see description below). Staff of Research Triangle Institute International, an independent, nonprofit research organization commissioned to conduct the NSDUH study funded by the Substance Abuse and Mental Health Administration (SAMHSA), obtained informed consent from participants [33]. Verbal informed consent was obtained from the parents/guardians of adolescents aged 12-17 years or from adolescents aged 17 years who were living independently [33]. Verbal informed assent was obtained from adolescents aged 12-17 living with their parents [33]. A more detailed explanation about the process of obtaining informed assent from adolescents can be found in the NSDUH Field Interviewer Manual (pages 232-236) [33]. Briefly, staff used an approved script to inform the parent/guardian that their child has been selected for the study, provided details about the study and informed them that their child’s participation is voluntary. Once parental permission was granted, the staff confirmed that an adult will be present for the entire duration of the interview with the child. Although an adult must be present in the house during the interview, the interview with the child is still held in a private space to ensure confidentiality. Informed assent is obtained from the child using an approved script. The child is informed that the study is voluntary, that their responses are confidential, and that they will be compensated with $30 after completing the interview. A description of the study procedures is then described to the child. Interview proceeds only after informed assent is obtained from the child.

### Sample population

This study used data from the publicly available 2019 NSDUH dataset [34]. The NSDUH is a national survey administered each year to approximately 70,000 US civilians aged 12 years and older. It employs a complex, multistage probability sampling design to allow for a representative selection of the U.S. population that collects national- and state-level data concerning tobacco, alcohol, and other drug use as well as mental health. Individuals who are active members of the military, hold no household address, or reside in institutional group quarters such as prisons, nursing homes, treatment centers, and hospitals are excluded from survey participation. The present study conducted a cross-sectional secondary analysis of questionnaire data collected from the 2019 NSDUH. This year was chosen due to concern that data from subsequent years would likely have been influenced by the COVID-19 pandemic. Inclusion criteria were participants between 12-17 years of age (N=13,397) who responded to questions about perception of risk of harm from weekly binge drinking (N=13,133). From this sample, we excluded participants who reported a history of binge drinking (N=1,263) given that this was a cross-sectional analysis and including participants with binge drinking, which lies distal to perception of risk of harm, is likely to cloud any existing relationship between extracurricular activities and perception of risk of harm. Thus, the final analytical sample consisted of 11,870 adolescents.

### Perceived risk of harm from weekly binge drinking (dependent variable)

The primary outcome variable was perceived risk of harm from weekly binge drinking, which was assessed through the question, “How much do people risk harming themselves physically and in other ways when they have five or more drinks of an alcoholic beverage once or twice a week?” [1,35–40]. Survey respondents were presented with four choices ranging from no risk to great risk, which we dichotomized into two categories for the purpose of this analysis: no risk of harm weekly binge drinking (“no risk”) and any risk of harm from weekly binge drinking (“great risk”; “moderate risk” “slight risk”) coded as 0/1. This dichotomization is consistent with previous studies that utilized the NSDUH to explore risk perception of marijuana use [41,42] and the team’s consideration that any perception of risk from binge drinking is favorable to perceiving no risk from this problematic behavior. S1 Appendix includes all variables from the 2019 NSDUH dataset used in this study.

### Participation in extracurricular activities (independent variables)

The NSDUH 2019 dataset contains four questions (corresponding to four different activity-types) asking respondents to report the number of activities they had participated in during the past 12 months (e.g., During the past 12 months, in how many different kinds of school-based activities, such as team sports, cheerleading, choir, band, student government, or clubs, have you participated?). Options included: “None,” “One,” “Two,” or “Three or More.” The dataset differentiates the four types of extracurriculars as 1) school-based [“such as team sports, cheerleading, choir, band, student government, or clubs”]; 2) community-based [“such as volunteer activities, sports, clubs, or groups”]; 3) faith-based [“such as clubs, youth groups, Saturday or Sunday school, prayer groups, youth trips, service or volunteer activities”]; and 4) other-type [“such as dance lessons, piano lessons, karate lessons, or horseback riding lessons”]. Since there were four activity-types, each with responses that ranged from 0 to 3 or more, the total possible range of number of activities spanned from “0” to “12 or more” across all types of activities. For the purposes of this study, we captured extracurricular participation in three distinct ways. First, we created a binary variable (none vs. 1 or more) where adolescents who reported no involvement in any extracurricular activity, regardless of activity type were coded as 0 and adolescents with involvement in one or more activity were coded as 1. Second, to explore the influence of different types of activities on perception of risk of harm from binge drinking, we created a binary variable (none vs. 1 or more) for each type of activity (school, community, faith, other). Lastly, to examine whether the number of activities influence risk perception, we examined the level of involvement in extracurricular activity (0= “None,” 1= “One,” 2= “Two,” or 3= “Three or more.”) in each activity type (school, community, faith, other).

### Covariates

Other variables to be considered in the study were selected based on the health belief model and the socio-ecological model, their associations with both the independent and dependent variables, as well as from existing studies of similar scope [43,44]. As such, the following demographic variables were included: age in years (12-13, 14-15, 16-17); sex at birth (female, male); race/ethnicity (non-Hispanic white, non-Hispanic black, Hispanic, other race); total family income (less than $20,000, $20,000-$49,999, $50,000-$74,999, $75,000 and above); and rural/urban residence (large metropolitan, small metropolitan, non-metropolitan). There is strong evidence to suggest that poor mental health is related to an individual’s assessment of risk and their involvement in extracurricular activities [2,30,45]. Therefore, whether the adolescent had reported a major depressive episode in the last year (yes/no) was included as a covariate. Similarly, the overall support provided to adolescents by their parent/guardian has been linked to their participation in extracurriculars along with their perception of risk [46,47]. As such, we created a parental support composite variable based on the responses to four questions assessing various aspects of this construct (e.g., “During the past 12 months, how often did your parents provide help with your homework when you needed it?”). Responses to these questions were “always”, “sometimes”, “seldom”, “never”. Participants who responded, “always or sometimes” to all four questions were coded as “high parental support”; otherwise, they were deemed “medium-to-low parental support.” This parental support construct has been used in a similar paper using the NSDUH survey data [44].

### Data analysis

All analyses were conducted in Stata Version 18.0 [48] and survey weights were used to account for the complex sampling design. The final analytical sample consisted of 11,870 eligible adolescents. None of the demographic data had any missing data as we used the imputed variables provided in the NSDUH dataset [35]. Data for depression and parental support were missing for 359 (3.02%) and 731 (6.16%) adolescents, respectively. Data for each extracurricular activity were missing as follows: school (n=106, 0.89%), community (n=165, 1.39%), faith (n=199, 1.68%), other (n=137, 1.15%). Missing data were imputed using Multiple Imputation by Chained Equations [49]. Frequencies and percentages were used to describe the sample across study characteristics. Bivariate regression analyses were used to examine differences between adolescents who perceived weekly binge drinking as harmful compared to adolescents who did not across all covariates. Bivariate and multivariate logistic regression models were run to test the unadjusted and adjusted associations between participation in extracurricular activities and perceived risk of harm from weekly binge drinking. Specifically, we ran separate models for activity participation measured as a binary variable regardless of activity type (none vs. 1 or more), activity participation measured as a binary variable (none vs. 1 or more) separated by activity type (school, community, faith, other), and activity participation measured as a ordinal variable (0, 1, 2, 3+ activities) across each activity-type (school-based, community-based, faith-based, and other-type extracurricular activities). For the latter two analyses examining each activity type, we conducted four different regression models that each contained only one type of activity (school, community, faith or other) to avoid multicollinearity since there is the possibility that adolescents who participate in one type or activity may be likely to participate in other types of extracurricular activity. All multivariate logistic regression models included relevant covariates (demographic factors, depression, and parental support). Both unadjusted (OR) and adjusted odds ratios (aOR) were reported along with their respective 95% confidence intervals (95% CI).

## Results

### Sample characteristics

Of the 11,870 eligible adolescents, approximately half in this sample identified as male (51.52%), non-Hispanic white (50.52%), and had a household income of $75,000 or more (45.03%) (See Table 1). Thirty-five percent of the sample were aged 12-13 years, with 35% aged 14-15 years and 30% aged 16-17 years. Most adolescents reported participating in at least one activity of any kind (91.69%). Over half of adolescents reported participating in two or more school-based activities (56.59%) while 47.53%, 36.21% and 17.83% participated in two or more community-based, faith-based or other activities respectively. About 4% of adolescents in the sample endorsed perceiving no risk of harm from weekly binge drinking.

**Table 1:** Demographics of adolescents by perception of risk of harm from binge drinking.

	Total sample (n=11,870)	Perception of risk of harm from binge drinking (Weighted %)		F (p-value)*
		Perceived no risk (n=503)	Perceived any risk (n=11,367)	
**Age** N (%)				
12-13	4,122 (35.04%)	40.99%	34.80%	12.31(0.001)^**^
14-15	4,140 (34.96%)	36.65%	34.89%	
16-17	3,608 (30%)	22.35%	30.31%	
**Sex/Gender** N (%)				
Male	6,150 (51.52%)	63.70%	51.02%	14.05(0.000)^***^
Female	5,720 (48.48%)	36.30%	48.98%	
**Race / Ethnicity** N (%)				
Non-Hispanic White	5,983 (50.52%)	40.97%	50.92%	9.16(0.004)^**^
Non-Hispanic Black	1,640 (13.99%)	18.81%	13.79%	
Hispanic	2,837 (24.97%)	28.79%	24.81%	
Other	1,410 (10.51%)	11.43%	10.47%	
**Total Household Income** N (%)				
$75,000 or more	5,009 (45.03%)	31.35%	45.59%	4.85(0.032)^*^
$50,000 - $74,999	1,797 (14.62%)	12.09%	14.72%	
$20,000 - $49,999	3,286 (26.4%)	30.67%	26.23%	
Less than $20,000	1,778 (13.95%)	25.90%	13.46%	
**Metro** N (%)				
Large metro	5,325 (55.05%)	53.90%	55.10%	0.06(0.806)
Small metro	4,234 (30.78%)	34.13%	30.64%	
Non-metro	2,311 (14.18%)	11.97%	14.27%	
**Depression** N (%)				
No	9,828 (85.66%)	89.30%	85.51%	3.19(0.081)
Yes	1,683 (14.34%)	10.70%	14.549%	
**Parental Support** N (%)				
Low parental support	3,873 (35%)	40.17%	34.86%	2.60(0.116)
High parental support	7,266 (65%)	59.83%	65.14%	
**Participation in any extracurricular activity** N (%)				
** **None	1,033 (8.31%)	16.55%	7.88%	30.93(0.000)^***^
1 or more activities	11,000 (91.69%)	83.45%	92.13%	
**School-based activity involvement** N (%)				
** **None	2,017 (17.07%)	29.44%	16.61%	21.34(0.000)^***^
1 activity	3,056 (26.34%)	25.21%	26.40%	
2 activities	3,092 (26.47%)	23.02%	26.57%	
3+ activities	3,599 (30.12%)	22.33%	30.41%	
**Community-based activity involvement** N (%)				
** **None	3,003 (25.56%)	36.31%	25.19%	12.80(0.001)^***^
1 activity	3,153 (20.98%)	25.62%	26.91%	
2 activities	2,493 (20.98%)	17.52%	21.11%	
3+ activities	3,056 (26.55%)	20.55%	26.79%	
**Faith-based activity involvement** N (%)				
** **None	5,089 (42.37%)	51.16%	42.08%	14.13(0.000)^***^
1 activity	2,494 (21.42%)	19.99%	21.47%	
2 activities	1,497 (12.61%)	16.26%	12.44%	
3+ activities	2,591 (23.6%)	12.59%	24.00%	
**Other activity involvement** N (%)				
** **None	7,367 (61.82%)	67.13%	61.61%	3.17(0.082)
1 activity	2,332 (20.36%)	16.49%	20.52%	
2 activities	935 (7.99%)	9.51%	7.92%	
3+ activities	1,093 (9.84%)	6.87%	9.95%	

Note:

* p<0.05,

** p<0.01,

*** p<0.001

Table 1 outlines the differences between adolescents who perceived weekly binge drinking as having no risk of harm compared to those who perceived any risk. Adolescents who perceived any risk of harm from weekly binge drinking were more likely to be female (F=14.05, *p*=0.000), 16-17 years old (F=12.31, *p*=0.001), Non-Hispanic White (F=9.16, *p*=0.004), and have a total household income of $75,000 or more (F=4.85, *p*=0.032). Additionally, adolescents who participated in any extra-curricular activity compared to those that did not participate were significantly more likely to perceive risk of harm from binge drinking weekly (F=30.93, *p*=0.000). Across school-based, community-based, and faith-based types of extracurricular activities, adolescents who were involved in one or more extracurricular activity were more likely to perceive that weekly binge drinking as risky (*School* F=21.34, p=0.000; *Community* F=12.80, *p*=0.001; *Faith* F=14.13, *p*=0.000). Participating in other types of activities was not significantly associated with perception of risk of harm.

### Associations between participation in any extracurricular activity and perception of risk of harm from weekly binge drinking

Table 2 presents the results of both the unadjusted and adjusted logistic regressions used to examine the associations between participating in any extracurricular activity regardless of type and perception of harm from binge drinking. Results show that adolescents who participate in at least one type of extracurricular activity have more than twice the odds of reporting that weekly binge drinking is harmful. This association remained significant after controlling for potential confounders (aOR = 1.86, 95% CI: 1.39, 2.49). In addition, being female and older (16-17 years) was associated with perceiving binge drinking as harmful while having a household income of less than $50,000 was associated with reduced likelihood of perceiving binge drinking as harmful (See Table 2).

**Table 2:** Participation in extracurricular activities and perception of risk of harm from binge drinking.

	Involvement in any extracurricular activity	
**Unadjusted**	**Adjusted**
**Number of activities**		
None	1	1
1 or more activities	2.32 [1.71, 3.15]***	**1.86 [1.39, 2.49]** ^***^
**Sex/ Gender**		
Male		1
Female		**1.58 [1.21, 2.07]** ^**^
**Age**		
12-13		1
14-15		1.13 [0.91, 1.41]
16-17		**1.67 [1.26, 2.21]** ^**^
**Race/ Ethnicity**		
Non-Hispanic White		1
Non-Hispanic Black		0.79 [0.59, 1.05]
Hispanic		0.91 [0.70, 1.20]
Other		0.79 [0.57, 1.09]
**Total Household Income**		
$75,000 or more		1
$50,000 - $74,999		0.86 [0.55, 1.37]
$20,000 - $49,999		**0.65 [0.48, 0.88]** ^**^
Less than $20,000		**0.41 [0.29, 0.58]** ^***^
**Metro**		
Large metro		1
Small metro		0.91 [0.71, 1.17]
Non-metro		1.22 [0.80, 1.86]
**Depression**		1.29 [0.86, 1.94]
**Parental Support**		1.32 [0.97, 1.78]

Note:

* p<0.05,

** p<0.01,

*** p<0.001

### Associations between participation in different types of extracurricular activity and perception of risk of harm from weekly binge drinking

Table 3 shows results of the association between different types of extracurricular activity (school, community, faith and other) and perception of harm from weekly binge drinking. Unadjusted analyses show that compared to adolescents who reported no participation in any extracurricular activity, those who reported one or more activities were significantly more likely to perceive that weekly binge drinking was harmful across all types of activities (Table 3). However, this association was sustained in the adjusted analyses for school, community and faith-based activities (*school* aOR: 1.82, 95% CI: 1.40, 2.38; *community* aOR: 1.40, 95% CI: 1.09, 1.79; *faith* aOR: 1.33, 95% CI: 1.03, 1.72). Like the first model (See Table 2), age, gender and income were associated with perception of risk of harm from binge drinking with older age and female sex being positively associated with higher perception of risk while lower income was associated with lower perception of risk (See Table 3).

**Table 3:** Association between types of extracurricular activity and perception of risk of harm: unadjusted and adjusted regression.

Variable	School-based		Community-based		Faith-based		Other	
Unadjusted	Adjusted	Unadjusted	Adjusted	Unadjusted	Adjusted	Unadjusted	Adjusted
**Number of activities**								
None	1	1	1	1	1	1	1	1
1 or more activities	**2.09 [1.62, 2.71]**	**1.82 [1.40, 2.38]**	**1.69 [1.31, 2.18]**	**1.40 [1.09, 1.79]**	**1.44 [1.11, 1.88]**	**1.33 [1.03, 1.72]**	**1.27 [1.04, 1.56]**	1.08 [0.86, 1.34]
**Sex/Gender**								
Male		1		1		1		1
Female		**1.59 [1.22, 2.07]**		**1.60 [1.22, 2.08]**		**1.59 [1.22, 2.08]**		**1.61 [1.22, 2.11]**
**Age**								
12-13		1		1		1		1
14-15		1.12 [0.90, 1.40]		1.11 [0.89, 1.39]		1.12 [0.90, 1.40]		1.12 [0.89, 1.40]
16-17		**1.66 [1.26, 2.20]**		**1.63 [1.22, 2.17]**		**1.67 [1.26, 2.21**]		**1.65 [1.24, 2.19]**
**Race/ Ethnicity**								
Non-Hispanic White		1		1		1		1
Non-Hispanic Black		0.79 [0.59, 1.05]		0.79 [0.59, 1.05]		0.78 [0.58, 1.04]		0.80 [0.60 1.07]
Hispanic		0.92 [0.70, 1.21]		0.91 [0.69, 1.20]		0.91 [0.70, 1.19]		0.90 [0.69, 1.19]
Other		0.77 [0.56, 1.07]		0.79 [0.57, 1.09]		0.80 [0.58, 1.10]		0.79 [0.58, 1.09]
**Total Household** Income								
$75,000 or more		1		1		1		1
$50,000 - $74,999		0.87 [0.55. 1.39]		0.90 [0.55. 1.37]		0.86 [0.54. 1.36]		0.85 [0.54 1.36]
$20,000 - $49,999		**0.65 [0.48, 0.88]**		**0.65 [0.48, 0.88]**		**0.63 [0.46, 0.86]**		**0.62 [0.46, 0.85]**
Less than $20,000		**0.42 [0.29, 0.59]**		**0.41 [0.29, 0.58]**		**0.39 [0.28, 0.56]**		**0.38 [0.27, 0.55]**
**Metro**								
Large metro		1		1		1		1
Small metro		0.90 [0.70, 1.16]		0.90 [0.70, 1.15]		0.89 [0.70, 1.15]		0.89 [0.69 1.15]
Non-metro		1.20 [0.79, 1.83]		1.21 [0.80, 1.85]		1.18 [0.78, 1.80]		1.21 [0.79, 1.84]
**Depression**								
No		1		1		1		1
Yes		1.30 [0.87, 1.94]		1.29 [0.86, 1.92]		1.31 [0.87, 1.96]		1.29 [0.86, 1.93]
**Parental Support**								
Low parental Support		1		1		1		1
High parental support		1.32 [0.97, 1.80]		1.32 [0.97, 1.80]		1.33 [0.99, 1.79]^#^		**1.36 [1.01, 1.84]**

Bold (p<0.05).

### Associations between number of extracurricular activity and perception of risk of harm from weekly binge drinking across different activity types

Unadjusted analyses revealed that participating in any number of school-based or community-based activities (1 activity, 2 activities, or 3+ activities) was significantly associated with having a higher perception of risk of harm from binge drinking, as well as participating in 3 or more faith-based activities or one other activity type (see Table 4). After adjusting the model for relevant covariates, participating in any number of school-based activities remained significant (aOR 1 activity = 1.75, 95% CI: 1.29, 2.37, aOR 2 activities = 1.83, 95% CI:1.32, 2.53, aOR 3+ activities = 1.91, 95% CI:1.35, 2.75), However, this effect was only seen for participating in either one or two community-based activities but not for three activities, although the p-value was 0.06 for three or more activities (aOR 1 activity = 1.35, 95% CI: 1.02, 1.79, aOR 2 activities = 1.47, 95% CI:1.06, 2.02, aOR 3+ activities = 1.41, 95% CI: 0.98, 2.02). Similarly, adjusted analysis was significant for only for participating in three or more faith-based activities, (aOR 3+ activities = 2.07, 95% CI: 1.48, 2.88).

**Table 4:** Association between levels of extracurricular activity and risk perception of binge drinking across different activity types.

Variable	School-based		Community-based		Faith-based		Other	
	Unadjusted	Adjusted	Unadjusted	Adjusted	Unadjusted	Adjusted	Unadjusted	Adjusted
**Level of involvement**								
None	1	1	1	1	1	1	1	1
1 activity	**1.86 [1.38, 2.50]**	**1.75 [1.29, 2.37]**	**1.51 [1.15, 2.00]**	**1.35 [1.02, 1.79]**	1.31 [0.87, 1.96]	1.24 [0.83, 1.85]	**1.36 [1.02, 1.80]**	1.16 [0.86, 1.56]
2 activities	**2.05 [1.49, 2.81]**	**1.83 [1.32, 2.53]**	**1.74 [1.24, 2.42]**	**1.47 [1.06, 2.02]**	0.93 [0.63, 1.37]	0.88 [0.60, 1.30]	0.91 [0.61, 1.35]	0.77 [0.51, 1.18]
3+ activities	**2.41 [1.69, 3.45]**	**1.91 [1.35, 2.75]**	**1.88 [1.32, 2.70]**	1.41 [0.98, 2.02]*	**2.32 [1.65, 3.25]**	**2.07 [1.48, 2.88]**	1.58 [0.94, 2.65]	1.30 [0.77, 2.20]
**Sex/Gender**								
Male		1		1		1		1
Female		**1.58 [1.22, 2.06]**		**1.59 [1.22, 2.09]**		**1.56 [1.20, 2.03]**		**1.60 [1.22, 2.10]**
**Age**								
12-13		1		1		1		1
14-15		1.12 [0.90, 1.40]		1.11 [0.88, 1.39]		1.15 [0.92, 1.43]		1.13 [0.90, 1.43]
16-17		**1.66 [1.26, 2.20]**		**1.63 [1.22, 2.16]**		**1.69 [1.27, 2.56]**		**1.67 [1.25, 2.23]**
**Race / Ethnicity**								
Non-Hispanic White		1		1		1		1
Non-Hispanic Black		0.79 [0.59, 1.05]		0.79 [0.59, 1.05]		0.79 [0.59, 1.06]		0.80 [0.60 1.07]
Hispanic		0.92 [0.70, 1.21]		0.91 [0.69, 1.20]		0.93 [0.71, 1.22]		0.91 [0.69, 1.19]
Other		0.77 [0.56, 1.07]		0.79 [0.57, 1.09]		0.80 [0.59, 1.10]		0.80 [0.58, 1.10]
**Total Household Income**								
$75,000 or more		1		1		1		1
$50,000 - $74,999		0.88 [0.56. 1.38]		0.88 [0.56. 1.37]		0.87 [0.55. 1.37]		0.85 [0.54 1.36]
$20,000 - $49,999		**0.65 [0.48, 0.88]**		**0.65 [0.48, 0.88]**		**0.64 [0.47, 0.88]**		**0.62 [0.46, 0.85]**
Less than $20,000		**0.42 [0.30, 0.60]**		**0.41 [0.29, 0.58]**		**0.39 [0.28, 0.56]**		**0.39 [0.27, 0.55]**
**Metro**								
Large metro		1		1		1		1
Small metro		0.90 [0.70, 1.16]		0.90 [0.70, 1.15]		0.89 [0.69, 1.15]		0.89 [0.69 1.15]
Non-metro		1.20 [0.79, 1.82]		1.21 [0.80, 1.85]		1.16 [0.76, 1.76]		1.21 [0.79, 1.85]
**Depression**								
No		1		1		1		1
Yes		1.30 [0.86, 1.94]		1.29 [0.86, 1.93]		1.30 [0.87, 1.95]		1.23 [0.86, 1.92]
**Parental Support**								
Low parental support		1		1		1		1
High parental support		1.32 [0.97, 1.80]		1.32 [0.97, 1.80]		**1.34 [1.00, 1.80]**		**1.37 [1.01, 1.84]**

Bold (p<0.005); *p=0.06

Similar to the first two models, additional significant findings emerged from the multivariate logistic regression model. Across all types of extra-curricular activities, females were more likely to endorse a high perception of risk of harm from binge drinking compared to males (aOR *school* = 1.58, 95% CI: 1.22, 2.06, aOR *community* = 1.59, 95% CI: 1.22, 2.09, aOR *faith* = 1.56, 95% CI: 1.20, 2.03, aOR *other* = 1.60, 95% CI: 1.22, 2.10). Additionally, there was an effect of age on risk perception across types of extra-curricular activities. Compared to 12–13-year-olds, 16–17-year-olds were more likely to endorse a higher risk perception (aOR *school* = 1.66, 95% CI: 1.26, 2.20, aOR *community* = 1.63, 95% CI: 1.22, 2.16, aOR *faith* = 1.69, 95% CI: 1.27, 2.56, aOR *other* = 1.67, 95% CI: 1.25, 2.23). Compared to those with a total household income of $75,000 or more, those with less than $20,000 and between $20,000-$49,000 had reduced odds of endorsing binge drinking as risky across all extra-curriculars (see Table 4). Parental support was significantly associated with increased risk perception from binge drinking in the faith-based activities and other activity models but not in the school-based or community-based activities models. Race/ethnicity, metropolitan areas, and depression were not significantly associated with perception of risk of harm from binge drinking across all models.

## Discussion

Using a large, nationally representative dataset with US adolescents aged 12-17, this study examined whether participation in extracurricular activities, along with type and number of activities, were associated with perception of risk of harm from binge drinking. Results from the present study indicate that adolescents who engaged in at least one extracurricular were almost 86% more likely to report that weekly binge drinking presented some risk compared to their nonparticipating counterparts. The type of activity seemed to influence this perception, as the odds of risk perception were higher by 82%, 40% and 33% for school, community and faith-based activities, respectively. When examining the number of activities in each type of activity, adolescents who participated in one, two, or three or more school-based activities were more likely to perceive binge drinking as harmful with increasing odds by number of activities. This pattern was different from community-based and faith-based activities. Adolescents who participated in one or two community-based activities, but not three or more activities, were 35% and 47% more likely to endorse that weekly binge drinking was harmful. In contrast, only adolescents who reported participating in three or more faith-based activities were significantly more likely to endorse weekly binge drinking as harmful, with two times the odds compared to adolescents who reported not participating in any faith-based activities.

To date, research examining the impact of extracurricular activities and alcohol use has focused predominantly on investigating school-based activities [50–53] or religious participation [54–56]. Most of these studies have revealed that the impact of these activities on alcohol use may vary by several factors (e.g., grade level, sport type, intensity (varsity vs. intramural), coach involvement, depth of spirituality), suggesting different mechanistic pathways [50,51,53,54,57–59]. We contribute to the literature by comparing multiple types of extracurricular activities, and by focusing not directly on problematic alcohol use or binge drinking but on an important antecedent: the perception of risk of harm from binge drinking.

Previous studies examining the association between extracurricular activities and perception of risk of harm from substances have centered around cannabis [41] or tobacco use [44]. This study extends the existing literature by providing the first examination focused on binge drinking. Our findings align with previous studies that found participation in any extracurricular activity to be associated with an increased perception of risk from cannabis and tobacco use. Both studies modeled extracurricular activity as a binary variable and did not account for levels of involvement or variations in activities types [41,44]. In addition, the study on tobacco use focused exclusively on school-based activities [44]. To build on this, we examined both the types and number of activities.

Participation in one or more school, community, and faith-based activities was significantly associated with adolescents’ perception of weekly binge drinking as harmful, suggesting that extracurricular involvement confers a protective effect regardless of activity type. Given that this is a cross-sectional study, these findings are inconclusive. Future longitudinal studies are needed to examine whether participation in extracurricular activities increases risk perception, which subsequently reduces future binge drinking among adolescents. Several factors may explain our finding that participating in extracurricular activities associated with enhanced risk perception of binge drinking. Engagement with these activities typically demands time, discipline, motivation, interactions with peers, and supervision, all of which may contribute to the shaping of key antecedents of alcohol-related risk perception [60]. Future studies are also needed to further elucidate whether these factors potentially mediate the association between extracurricular activities and perception of risk of harm from binge drinking.

Although participating in any school-, community-, or faith-based activities was associated with increased risk perception, the number of activities also appeared to play a role, suggesting different mechanistic effects across these types of activities. Adolescents who participated in one, two, or three or more school-based activities were 75%, 83% and 91% more likely to perceive binge drinking as harmful, respectively, compared to adolescents who did not participate in any, suggesting a possible dose effect with school-based activities. However, community-based activities were significant for only one or two activities and faith-based activities were significant for only three or more activities. The influence of increasing number of school-based activities on the perception of risk of harm from binge drinking may be reflective of students having more of their time spent engaged in more athletic activities where their performance is negatively impacted by binge drinking [61]. As colleges increasingly consider extracurricular activities in their selection process, students engaged in these activities may be more likely to reflect on the harm that binge drinking could have on both their performance and future college prospects. Moreover, participation in structured, productive activities often involve education about the consequences of substance use, thereby raising students’ awareness and perception of harm associated with these behaviors. Other studies have also suggested that the influence of extracurricular participation on risk perception may be modified by the attitudes and behaviors of the other students on the team; students surrounded by peers who have unfavorable attitudes toward substance use are more likely to develop those attitudes themselves and perceive that alcohol use as risky and in turn less likely to use alcohol [62]. Additionally, participation may require adherence to formal or informal agreements prohibiting illicit behavior, possibly enhancing adolescents’ knowledge of substances and their associated risks [63]. The loss of significance in the adjusted models for three or more community-based activities seems to suggest that greater than two community-based activities confer no additional benefit on risk perception of binge drinking and that the effect of number of community-based activities on risk perception may better explained by additional factors or separate pathways. Spirituality and religious involvement are frequently linked to lower rates of substance use among youth [56,57] and has been shown to be associated with reduced likelihood of binge drinking among adolescents and young adults [54,58]. The higher perception of risk of harm among adolescents who participate in three or more faith-based activities could be attributed in part to a deeper spirituality [54] and commitment to religious beliefs or greater exposure to messages that discourage unhealthy behaviors, including binge drinking. Within the context of the present study, it remains unclear whether “other” types of activities can serve as strategies in preventing in adolescent substance use. It is possible that categories such as “other” activities are too broad to detect significant differences in the NSDUH sample. The types of activities reflected in this category (e.g., piano lessons or horseback riding) typically rely heavily on parental motivation or support and may be more likely to be occurring in addition to other school- or community-based activities, both of which may exert a greater effect on the perception of risk of harm. These activities are also more likely to occur on an individual basis rather than in teams and as such coaches may be less inclined to discuss the harms of alcohol use with an individual compared to a group setting, where the performance of an individual may be detrimental to the team’s achievements. Future studies are needed to elucidate the role of different types of extracurricular activities to clarify existing mechanistic or mediating factors that may then inform targeted preventive strategies.

Other important covariate patterns emerged from our final adjusted models. Across all models and the four activity types, sex/gender, age, and income were significantly associated with perception of risk of harm. Adolescents who were female or 16-17 years old were more likely to endorse binge drinking as risky. Gender differences in risk perception have been well documented: females are generally more cautious of environmental and health hazards than males, largely due to socialization, biological factors, and a greater propensity to focus on the possibility of negative outcomes [64,65]. As the adolescent brain develops, adolescents improve their ability to self-regulate, make informed decisions, and resist peer pressure [66]. In turn, this cognitive maturity may enable 16–17-year-old teenagers to perceive binge drinking as riskier than those who are 12-13 years. Lastly, adolescents from households with income less than $50,000 were more likely to perceive that binge drinking was not harmful compared to adolescents from higher-income households. This is an interesting paradox given that studies have repeatedly shown that adolescents from households with higher income are more likely to engage in problematic alcohol use or binge drinking [67,68], suggesting that the protective effect of socioeconomic status on risk perception may not translate to use. Greater access to financial and social resources may offset the perceived negative consequences of binge drinking, suggesting that risk perception of binge drinking may be an important but insufficient independent factor in predisposing to future binging among adolescents from high income households. Understanding how different segments of the adolescent population perceive the risk of binge drinking and the influence of other moderating and/or mediating factors is essential when considering effective prevention strategies and public health messaging.

While this study highlights the positive role of school-based activities in substance misuse prevention, not all schools offer these programs. Insufficient funding may limit school systems from allocating funds toward implementing and sustaining these initiatives. Additionally, several factors may affect an adolescent’s likelihood of engaging in other types of activities (community, faith, other). For example, engaging in other activities such as horse-back riding depends on financial means, while access to community-based activities may depend on where one lives. Participating in faith-based activities may depend on parents’ own engagement in faith-based activities. Given that over 90% of adolescents attend school [69] and spend a significant amount of their time in school, our study findings showing benefits from participating in even one extracurricular activity suggests considering policies that prioritize federal and state funding to expand the accessibility of these programs. On the other hand, schools may have other non-financial challenges that limit their ability to provide multiple extracurricular activities. The potential benefits of participating in faith- and community-based activities highlighted in this study suggest the need for a comprehensive approach to prevention programing that serves the unique interests and harnesses the existing resources of all children.

### Limitations and future research

The current study was subject to several limitations. The cross-sectional design of the NSDUH 2019 dataset limited our ability to infer whether participation in extracurricular activities is causally linked to an enhanced risk perception of binge drinking. Prospective experimental designs would be most effective in better understanding the direction of this relationship and to measure the long-term effect of these activities. Second, we cannot be certain that an increased perception of risk of harm from binge drinking translates to reduced alcohol use; however, there is evidence to suggest that a high-risk perception is a deterrent of future binge drinking [1]. Third, the way the NSDUH assesses perceived risk of harm may be confusing to some adolescents, particularly the phrase “harming themselves physically and in other ways” which may result in either an under- or overestimation of risk depending on the adolescent’s interpretation. However, this phrasing has been utilized widely in the field for measuring perceived risk from alcohol and other substances [37,39,40,44,70]. Fourth, the four broad categorization of extracurricular activities available in this dataset prevented additional examination into the potential differential impact of specific types of activities on perception of risk of harm. Further studies should consider including a detailed list of activities to capture the possibility that, even within specific settings (e.g., schools), certain activities offer distinct benefits. Fourth, there are additional confounding variables such as health literacy and cognitive ability that were not assessed in the dataset but are likely associated with participation in extracurricular activities and risk perception. Lastly, although this is a nationally representative sample of US adolescents, these findings lack generalizability to those excluded from the NSUH sample, such as adolescents who are homeless.

## Conclusion

Increasing perception of risk of harm from binge drinking may effectively reduce the rates of adolescent binge drinking. Using a nationally representative sample of US adolescents, the study reveals that participating in any extracurricular activity was associated with increased risk of harm from binge drinking regardless of whether this was a school-based, community-based or faith-based activity. Adolescents who participated in an increasing number of school-based activities were also more likely to perceive that binge drinking was harmful while impact of the number of activities was significant for one or two community-based activities and for three or more faith-based activities. Future studies are needed to uncover potential mediating factors accounting for this association as well as whether the protective effect of extracurricular activity on risk perception translates to reduced binge drinking. Overall, encouraging participation in extracurricular activities appears beneficial for adolescents suggesting a need for a concerted effort to ensure equitable resources to develop these opportunities in schools and communities where adolescents reside.

## Supporting information

S1 Appendix Variables from the 2019 National Survey on Drug Use and Health (NSDUH) Dataset. (DOCX)
