# Supplementary material for: Adolescent extracurricular activities and perception of risk of harm from binge drinking
Source: PLOS Ment Health. 2025 Apr 9;2(4):e0000278. doi: 10.1371/journal.pmen.0000278 (PMC12162072; doi:10.1371/journal.pmen.0000278)
Supplement: S1 Appendix — (DOCX) [file pmen.0000278.s001.docx]

**S1 Appendix**

**Variables from the 2019 National Survey on Drug Use and Health (NSDUH) Dataset**

**Variables used in the creation of main independent variables: *participation in extracurricular activities***

YESCHACT: During the past 12 months, in how many different kinds of school-based activities, such as team sports, cheerleading, choir, band, student government, or clubs, have you participated?

0 = None

1 = One

2 = Two

3 = 3 or more

YECOMACT: During the past 12 months, in how many different kinds of community-based activities, such as volunteer activities, sports, clubs, or groups have you participated?

0 = None

1 = One

2 = Two

3 = 3 or more

YEFAIACT: During the past 12 months, in how many different kinds of church or faith-based activities, such as clubs, youth groups, Saturday or Sunday school, prayer groups, youth trips, service or volunteer activities have you participated?

0 = None

1 = One

2 = Two

3 = 3 or more

YEOTHACT: During the past 12 months, in how many different kinds of other activities, such as dance lessons, piano lessons, karate lessons, or horseback riding lessons, have you participated?

0 = None

1 = One

2 = Two

3 = 3 or more

**Variable used in the creation of the dependent variable: *perceived risk of harm from weekly binge drinking***

RSKBNGWK: How much do people risk harming themselves physically and in other ways when they have five or more drinks of an alcoholic beverage once or twice a week?

1 = No risk

2 = Slight risk

3 = Moderate risk

4 = Great risk

**Variables used in the creation of the following covariates:**

**AGE:**

AGE2: Recode – Final Edited Age

1= 12 years old

2= 13 years old

3= 14 years old

4= 15 years old

5= 16 years old

6= 17 years old

**SEX AT BIRTH:**

IRSEX: Imputation revised gender

1= Male

2=Female

**RACE/ETHNICITY:**

NEWRACE2 = RC-RACE/HISPANICITY RECODE (7 LEVELS)

1 = NonHisp White

2 = NonHisp Black/Afr Am

3 = NonHisp Native Am/AK Native

4 = NonHisp Native HI/Other Pac Isl

5 = NonHisp Asian

6 = NonHisp more than one race

7 = Hispanic

**TOTAL FAMILY INCOME:**

INCOME: TOTAL FAMILY INCOME RECODE

1 = Less than $20,000

2 = $20,000 - $49,000

3 = $50,000 - $74,999

4 = $75,000 or More

**RURAL/URBAN RESIDENCE:**

COUTYP4: COUNTY METRO/NONMETRO STATUS (2013 3-LEVEL)

1 = Large Metro

2 = Small Metro

3 = Nonmetro

**MAJOR DEPRESSIVE EPISODE:**

YMDEYR: PAST YEAR MAJOR DEPRESSIVE EPISODE (MDE)

1 = Yes

2 = No

**PARENTAL SUPPORT:**

PARCHKHW: PARENTS CHECK IF HOMEWORK DONE IN PST YR

1 = Always/Sometimes

2 = Seldom/Never

PARHLPHW: PARENTS HELP WITH HOMEWORK IN PST YR

1 = Always/Sometimes

2 = Seldom/Never

PRGDJOB2: PARENTS TELL YTH HAD DONE OOD JOB IN PST YR

1 = Always/Sometimes

2 = Seldom/Never

PRPROUD2: PARENTS TELL YTH PROUD OF THINGS DONE IN PST YR

1 = Always/Sometimes

2 = Seldom/Never
